# Inhibition of Poly(A)-binding protein with a synthetic RNA mimic reduces pain sensitization in mice

**DOI:** 10.1038/s41467-017-02449-5

**Published:** 2018-01-02

**Authors:** Paulino Barragán-Iglesias, Tzu-Fang Lou, Vandita D. Bhat, Salim Megat, Michael D. Burton, Theodore J. Price, Zachary T. Campbell

**Affiliations:** 1 School of Behavioral and Brain Sciences, University of Texas at Dallas, Richardson, TX 75080 USA; 2 Department of Biological Sciences, University of Texas Dallas, Richardson, TX 75080 USA

## Abstract

Nociceptors rely on cap-dependent translation to rapidly induce protein synthesis in response to pro-inflammatory signals. Comparatively little is known regarding the role of the regulatory factors bound to the 3′ end of mRNA in nociceptor sensitization. Poly(A)-binding protein (PABP) stimulates translation initiation by bridging the Poly(A) tail to the eukaryotic initiation factor 4F complex associated with the mRNA cap. Here, we use unbiased assessment of PABP binding specificity to generate a chemically modified RNA-based competitive inhibitor of PABP. The resulting RNA mimic, which we designated as the Poly(A) SPOT-ON, is more stable than unmodified RNA and binds PABP with high affinity and selectivity in vitro. We show that injection of the Poly(A) SPOT-ON at the site of an injury can attenuate behavioral response to pain. Collectively, these results suggest that PABP is integral for nociceptive plasticity. The general strategy described here provides a broad new source of mechanism-based inhibitors for RNA-binding proteins and is applicable for in vivo studies.

## Abstract (web summary)

Poly(A)-binding protein (PABP) is an RNA binding protein with translation function. Here, Barragán-Iglesias and colleagues devise an RNA mimic that inhibits PABP activity, and show that inhibitors can reduce animal’s pain response in vivo when injected locally.

## Introduction

Post-transcriptional gene control is a dominant theme in neuronal plasticity^1,2^. Messenger RNA (mRNA) possess two distinct structural features on opposing ends: a cap and a Poly(A) tail. Each structure serves as a molecular scaffold that nucleates the formation of dynamic multiprotein regulatory complexes^3–5^. These large assemblies enable signal-dependent control of protein synthesis. The cap-binding complex, consisting of eIF4F proteins, has emerged as a key player in pain sensitization^6–8^. Pain can be triggered by inflammation, nerve injury, and production of inflammatory cytokines (e.g., nerve growth factor (NGF) and interleukin 6 (IL-6)). NGF and IL-6 rapidly stimulate cap-dependent translation in nociceptors, resulting in long-term changes in excitability^8^. Far less is known regarding the regulatory impact of pro-inflammatory signals on regulation that occurs on the 3′ end.

Regulated cytoplasmic polyadenylation serves crucial roles in the developing nervous system and in the adult nervous system^9^. Moreover, synaptic plasticity can result in stimulation of factors that trigger addition of adenosines onto the 3′ end of mRNA^10–12^. The direct consequence of Poly(A) extension is increased binding of Poly(A)-binding proteins (PABPs)^13^. PABPs are master regulators of mRNA stability; their association with the Poly(A) tail protects the 3′ end from deadenylation and subsequent decay^14–16^. PABPs promote translation initiation through simultaneous associations with the Poly(A) tail and translation factors associated with the 5′ 7-methyl guanosine cap^13^. The interaction between eIF4G and PABP is essential for circularizing mRNA prior to eIF3-mediated recruitment of the 40S ribosomal subunit. RNA circularization is dictated by availability of PABPs, which is in turn controlled by the length of the Poly(A) tail. Despite recent evidence for PABP function in the central nervous system, little is known regarding the role of PABPs in induced plasticity^7^.

For many RNA-binding proteins, specificity is well established^17^. In principle, this information provides a means to generate RNA-based competitive inhibitors. However, a major complication of this approach is the ephemeral nature of RNA. RNA is rapidly degraded by exonucleolytic and endonucleolytic pathways. However, significant advances have been made in increasing RNA stability through the use of chemical modifications to the RNA 2′ hydroxyl group and the phosphodiester linkage^18,19^. These enhancements can increase RNA stability by an order of magnitude^20^. We hypothesize that the binding specificity of RNA-binding proteins in general can be used to guide the design of chemically stabilized RNA.

As a proof of concept, we examine the specificity of PABP using functional genomics to probe specificity in an unbiased way. Based on this information, we generate and characterize a chemically stabilized RNA substrate that binds to PABP with high specificity in vitro and impairs nascent translation in a PABP-dependent mechanism in cells. PABP is expressed throughout the peripheral nervous system and we target its function in mice in peripheral axons. We demonstrate that the effects of the RNA decoy on translation are specific to the initiation phase of translation and that axonal protein synthesis is impaired in nociceptor neurons. The Poly(A) SPOT-ON impairs pain sensitization in multiple models of tissue injury in vivo. Collectively, these experiments provide a guide for the rational design of RNA-binding protein inhibitors for mechanistic studies in cells or living animals.

## Results

### Unbiased analysis of PABP specificity

**Fig. 1:**
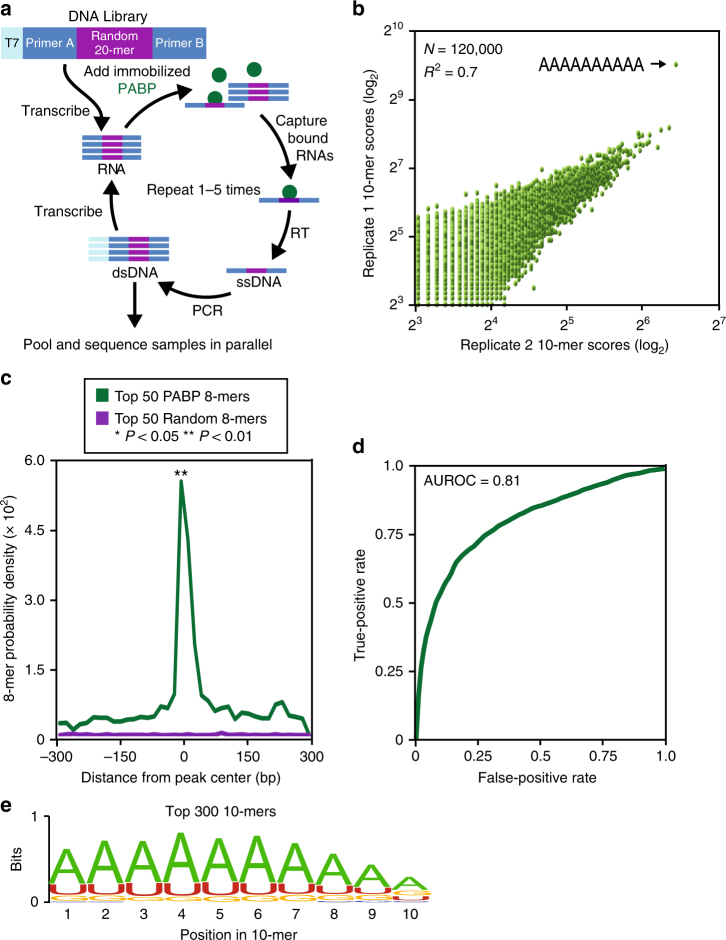
Unbiased assessment of PABP specificity and in vivo confirmation. **a** The SEQRS strategy begins with in vitro transcription of a DNA library containing a T7 primer (light blue), two constant regions (Primers a and b, dark blue), and a randomized 20-mer (purple). Following in vitro transcription, the library was incubated with PABP immobilized onto magnetic resin (green). RNA–protein complexes were isolated through wash steps and the bound RNAs were reverse transcribed. The T7 promoter was reattached through incorporation into a PCR primer and the process was repeated for five rounds prior to Illumina high-throughput sequencing. **b** Reproducibility of SEQRS. The most abundant 120,000 sequences for SEQRS replicates have a Pearson’s correlation coefficient of 0.7. The most enriched 10-mer sequence is an adenosine homopolymer and is indicated with an arrow. **c** Positions of the 50 most enriched 8-mer sequences from SEQRS for either PABP (green) or random sequences (purple) were calculated across known sites of PABP association outside of the Poly(A) tail in cells^25^. Enrichment scores were calculated based on the Mann–Whitney *U* test. **d** The area under the receiver operator curve is 0.81. **e** The sequence logo based on the top 300 10-mer sequences following SEQRS

Our experiments focus on the major cytoplasmic PABP isoform *PABPC1* (henceforth referred to as PABP) as it is the most abundant isoform based on high-throughput sequencing of the dorsal root ganglia (DRG) (Supplementary Fig. 1a)^21^. Furthermore, we were unable to detect a clear signal of the second most abundant isoform in the DRG by immunofluorescence (Supplementary Fig. 1b). We examined the specificity of PABP for all possible 10 base sequences using in vitro selection, high-throughput sequencing of RNA, and sequence specificity landscapes (SEQRS; Fig. 1a). This versatile approach has been successfully applied to RNA-binding proteins that recognize structured or linear elements and protein complexes^22–24^. PABP produced a highly reproducible pattern of enrichment (Fig. 1b). The most enriched sequence was an adenosine homopolymer. However, the diverse landscape of PABP that targets outside the Poly(A) tail suggests that interruptions in the Poly(A) sequence are tolerated in endogenous binding sites^25^. To determine if information obtained by SEQRS analysis of PABP in vitro values predicts the observed patterns of PABP occupancy in cultured mouse erythroleukemia cells, a model for PABP based on the top 50 8-mers was compared to a negative control with a similar compositional bias (Fig. 1c). The PABP model correctly identified genuine sites of occupancy in vivo (Wilcoxon–Mann–Whitney rank-sum test *P* < 0.003). To estimate the sensitivity and specificity of the PABP model, the bound sequences were used to estimate the area under the receiver operated curve (AU-ROC; Fig. 1d). The model performs well at discriminating between true positives relative to false positives (AU-ROC = 0.81). The repertoire of preferable PABP recognition sequences is apparent based on alignment of the top 300 10-mers which indicate a strong preference for A throughout the motif with a bias towards U followed by G at the first 9 positions (Fig. 1e). Position 10 has a slight preference for G over U. Based on these comprehensive measurements, we conclude that PABP is highly specific for sequences that are rich in adenosine with a preference toward adenosine homopolymers—a result consistent with known regulatory functions on the Poly(A) tail and elsewhere^16,26–29^.

### Design of a novel PABP inhibitor

As a novel means of competitively inhibiting PABP function, we applied our unbiased assessment of PABP specificity toward the development of specificity-derived competitive inhibitor oligonucleotides (SPOT-ON). We modified RNAs that are designed to bind to PABP in order to stabilize them in two key ways. First, to reduce the ability of the 2′ ribose hydroxyl to catalyze intramolecular cleavage, we incorporated 2′ *O*-methyl ribose modifications throughout the RNA^30^. Second, to reduce the activity of exonucleases in either direction, the 5′ and 3′ most base in the phosphodiester linkage was replaced with sulfur giving rise to terminal phosphorothioate bonds^31^. A minimum of 11–12 adenosines are required for high affinity binding to PABP^32^. Therefore, a compact 12 base RNA termed the Poly(A) SPOT-ON was generated as a potential competitive inhibitor with the composition A*AAAAAAAAAA*A (where * denotes phosphorothioate linkages). The Poly(A) SPOT-ON mimics the composition of the Poly(A) tail. As a key negative control, we used a random sequence with identical chemical configuration as before and designate this RNA as the scramble SPOT-ON (U*AACAAAAUAA*U).

**Fig. 2:**
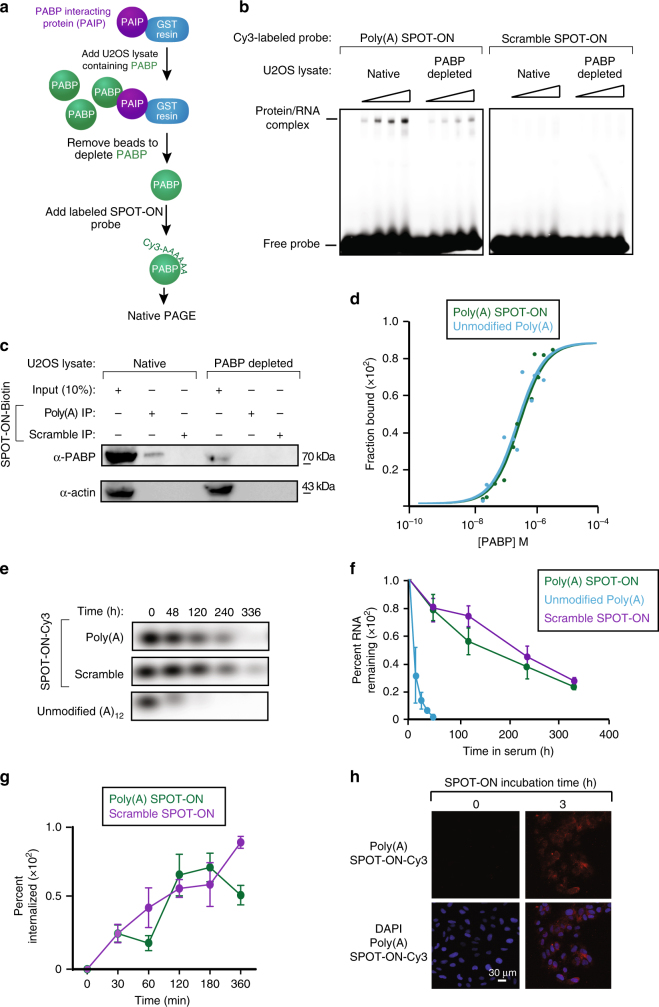
Characterization of the in vitro binding specificity of the Poly(A) SPOT-ON and cellular uptake. **a** The experimental approach for generation of PABP-depleted extracts consisted of immobilization of the PABP-interacting protein (PAIP, purple) onto resin (blue). Extracts containing PABP (green) were allowed to incubate and were aspirated resulting in loss of PABP. Cy3-labeled SPOT-ONs were added to total protein lysates and analyzed by electrophoretic mobility shift assay (EMSA). **b** EMSA assays. SPOT-ONs were incubated with either total protein lysate or PAIP-treated lysate and incubated at 0 °C for 40 min prior to separation by non-denaturing electrophoresis. The position of free probe and a single population of protein/RNA complex is indicated. This population is only observed in the Poly(A) SPOT-ON sample and is sensitive to PAIP depletion. The scramble SPOT-ON failed to shift a single population of proteins. **c** Pull-down experiments were conducted from lysates as prepared in **b**, but the SPOT-ON was generated with a biotin tag. Immunostaining is shown for either PABP or actin as a negative control. The Poly(A) SPOT-ON specifically associated with PABP in PABP containing lysates. **d** Equilibrium dissociation constants were determined by florescence anisotropy measurements of either unmodified adenosine dodecamer (blue) or the Poly(A) SPOT-ON (green). A modified version of the Michaelis–Menten equation was utilized to determine the equilibrium dissociation constants of either 261 ± 54 or 301 ± 41 μM for the 12 base unmodified or Poly(A) SPOT-ON RNAs, respectively. **e** Stability measurements of Cy3-labeled Poly(A) (green) or scrambled (purple) SPOT-ONs were determined in 10% FBS incubated at 37 °C and compared to a non-stabilized Poly(A) RNA (blue). **f** Quantification of **e**, percentage remaining is based on the initial intensity of RNA at time zero. *n* = 3. Data are plotted as mean ± s.e.m. **g** Cellular uptake of SPOT-ONs was determined based on imaging of U2OS cells for the Poly(A) and scrambled SPOT-ONs over time. *n* = 6. Data are plotted as mean ± s.e.m. **h** Sample data are shown for the Poly(A) SPOT-ON at time zero and after 3 h

We examined if the Poly(A) SPOT-ON binds to PABP in a series of in vitro experiments. In the first series of experiments, two extracts were prepared. We made use of an established protocol for efficient depletion of PABP by pre-incubation with immobilized PABP-interacting protein (PAIP) (Fig. 2a)^33^. As a negative control, a second extract containing PABP was mock depleted in parallel. Each lysate was incubated with either the Poly(A) SPOT-ON or the scramble SPOT-ON and subjected to native electrophoresis (Fig. 2b). We found a single clear band present in the Poly(A) SPOT-ON sample which is greatly reduced in intensity following PABP depletion (53%). Importantly, the negative control lacked clear binding to any species present in the whole cell extract. As an additional test for specificity, the SPOT-ONs were generated with 3′ biotin labels and again incubated with whole cell lysate. After allowing binding to proceed and performing numerous wash steps, we probed input and IP samples with antibodies for either PABP or actin (Fig. 2c). We found evidence for specific binding between the Poly(A) SPOT-ON which was diminished in PABP-depleted samples. Finally, equilibrium dissociation constants were determined by florescence polarization for PABP bound to either an unmodified 12 base Poly(A) sequence or the Poly(A) SPOT-ON (Fig. 2d). Non-linear least-squares regression analysis yielded *K*_d_ values of 261 ± 54 and 301 ± 41 nM for the unmodified or Poly(A) SPOT-ON, respectively. These results collectively argue that the Poly(A) SPOT-ON interacts with PABP with a high degree of specificity in vitro.

The stability of the SPOT-ONs was compared to unmodified RNA to determine if the modifications to the SPOT-ON enhanced stability. Indeed, the half-life of the unmodified RNA was approximately 18 h (Fig. 2e, f). Comparable measurements of the SPOT-ON indicate half-lives of >10 days. We also examined the cellular uptake of the SPOT-ONs in U2OS cells (Fig. 2g). The SPOT-ONs are efficiently taken up and are distributed throughout the U2OS cells after a 3-h period (Fig. 2h, Supplementary Fig. 2).

### The Poly(A) SPOT-ON reduces translation

**Fig. 3:**
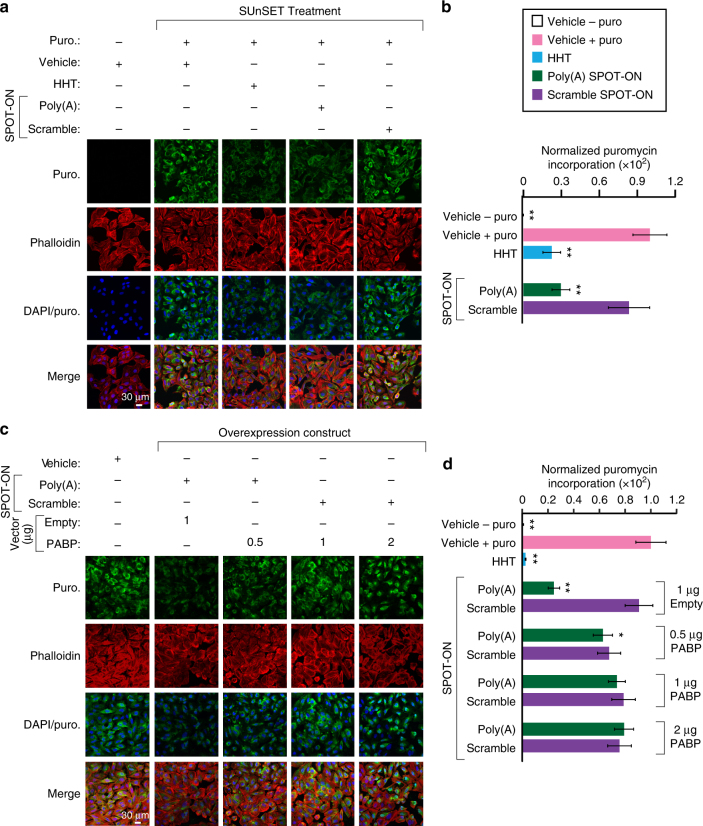
The Poly(A) SPOT-ON attenuates nascent protein synthesis. **a** SUnSET measurements in U2OS cells were conducted in the absence of puromycin to determine background levels of signal. Puromycin staining (green), phallodin (red), DNA (blue), and the merge between channels are arranged from top to bottom. As a positive control, puromycin and vehicle were used to determine the upper limit of translation. Both homoharringtonine (HHT) and the Poly(A) SPOT-ON robustly decrease protein synthesis, whereas the scrambled SPOT-ON failed to do so. **b** Quantification of **a**, empty boxes indicate no puromycin control, pink boxes are the positive control, blue boxed are homoharringtonine, green boxes are the Poly(A) SPOT-ON, and purple boxes are the scramble control. *n* = 15. **c** PABP overexpression rescues decreased protein synthesis caused by the Poly(A) SPOT-ON. Drug treatments consisted of either vehicle or SPOT-ON in the presence of an empty vector or overexpressed PABP. The amount of vector is indicated above the row of images. Markers are arranged as in **a**. **d** Quantification of **c**. *n* = 6. Columns represent measurements in the same manner as in **b**. *P < 0.05, **P < 0.01, significantly different from vehicle+puro group analyzed by one-way ANOVA followed by Bonferroni post hoc test. For all graphs shown in the figure, data are plotted as mean ± s.d

Using the non-radiometric surface sensing of translation Surface Sensing of Translation (SUnSET) approach, we measured nascent protein synthesis levels in U2OS cells (Fig. 3a, b)^34^. In this method, the structural analog of an aminoacyl-transfer RNA, puromycin, is used because it is readily incorporated into elongating polypeptides^35^. This causes termination of peptide elongation and release of the nascent peptide. The levels of puromycin can be visualized using a highly specific monoclonal antibody. In our experiments, we used a cytoskeletal marker for filamentous actin, phalloidin, as an internal control for differences in the number of cells in each image. As a key negative control, we excluded puromycin and observed little background signal. Inclusion of puromycin resulted in robust levels of translation. However, introduction of either homoharringtonine, an inhibitor of elongation, or the Poly(A) SPOT-ON reduced nascent protein synthesis by 77.6% and 70.4%, respectively (*F*_4, 72_ = 254, *P* < 0.0001; Fig. 3b). The scrambled SPOT-ON did not produce a significant effect.

To determine if the reduction in protein synthesis was due to inhibition of PABP, we transfected either an empty overexpression vector, pCDNA3, or a vector encoding full-length PABP (Fig. 3c, d). PABP expression was confirmed by immunoblotting (Supplementary Fig. 3, Supplementary Fig. 4). We found that the robust inhibition of protein synthesis caused by the Poly(A) SPOT-ON was ameliorated by PABP overexpression with the largest effects seen at high vector concentrations (*F*_10, 63_ = 180.1, *P* < 0.0001; Fig. 3d). Thus, PABP expression significantly increased protein synthesis in the presence of the Poly(A) SPOT-ON. Addition of transfection reagents non-specifically reduced protein synthesis by 10–20%. These changes are consistent but not significant relative to the untreated positive control. Following overexpression of PABP, the amount of nascent protein synthesis observed for the scramble and Poly(A) SPOT-ON is indistinguishable. This suggests strongly that PABP is the relevant cellular target of the SPOT-ON.

### The Poly(A) SPOT-ON impairs initiation

To further characterize the mechanism of action of the Poly(A) SPOT-ON, we made use of a modified version of SUnSET termed ribopuromycylation (RPM) to assay ribosome runoff^36–39^. Unlike SUnSET, cells are incubated with an irreversible inhibitor of elongation (emetine) and thus nascent chains are unable to dissociate from the ribosome and re-initiation is inhibited. Puromycylated proteins do not accumulate in monosomal fractions and sediment exclusively with heavy polysomes^37^. Thus, only a single round of translation is assayed through the use of puromycin immunofluorescence and normalized as before. We used this approach to differentiate what step in protein synthesis is impacted by the Poly(A) SPOT-ON through order of addition experiments.

**Fig. 4:**
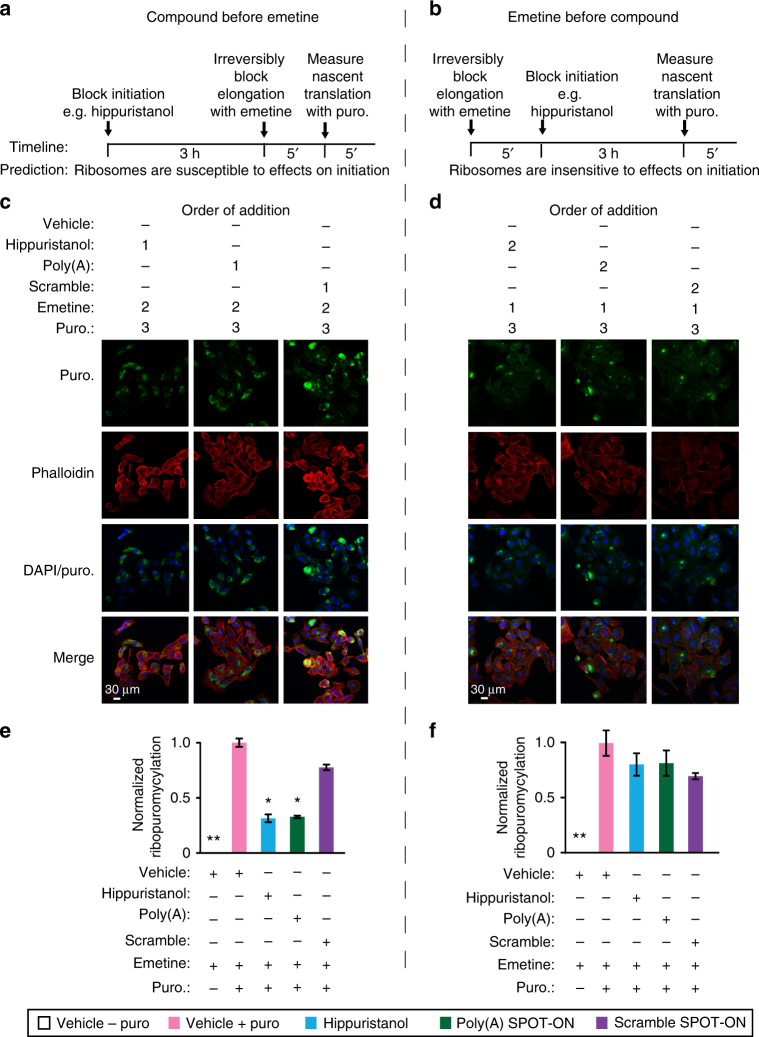
The Poly(A) SPOT-ON acts on initiation phase of protein synthesis. **a** In the first series of experiments, test compounds (e.g., hippuristanol) are added to cells and allowed to incubate prior to blockade of elongation with emetine. After 5 min puromycin is incorporated for a brief period of time. A predicted outcome of this experiment is that the ribosomes are susceptible to effects on initiation. **b** In a second series of experiments, elongation is blocked prior to initiation. Ribosomes are predicted to be insensitive to initiation inhibitors owing to prior arrest at a subsequent phase of translation (elongation). **c**, **d** Order of addition is indicated for either vehicle, hippuristanol, SPOT-ON RNAs, emetine, or puromycin. All samples receive emetine at the indicated time points (**a**, **b**). As before, staining is shown from top to bottom for puromycin (green), phallodin (red), DNA (blue), or a merge. **e** Quantification of **c**, empty boxes indicate no puromycin control, pink boxes are the positive control, blue boxes are hippuristanol, green boxes are the Poly(A) SPOT-ON, and purple boxes are the scramble control. Both hippuristanol and the Poly(A) SPOT-ON possess defective translation, whereas the scramble SPOT-ON does not. *n* = 6. **f** Quantification of **d**, where addition of emetine prior to test compounds fails to reveal significant differences for any of the test compounds. Columns represent measurements in the same manner as in **e**. *n* = 6. *P < 0.05, **P < 0.01, significantly different from vehicle + emetine + puro group analyzed by one-way ANOVA followed by Bonferroni post hoc test. For all graphs shown in the figure, data are plotted as mean ± s.d

We examined the effects of the eIF4A inhibitor hippuristanol as a key positive control for disruption of initiation of protein synthesis (Fig. 4a, b). We reasoned that by disrupting initiation prior to elongation, the final availability of ribosomes should reflect differences in initiation efficiencies. We added either hippuristanol, the Poly(A) SPOT-ON, or the scramble SPOT-ON prior to blocking elongation irreversibly with emetine and labeling ribosome-associated polypeptide chains with puromycin. We found that addition of either hippuristanol or the Poly(A) SPOT-ON significantly reduced RPM staining, whereas a vehicle or scramble SPOT-ON treatment did not (*F*_4, 19_ = 157.2, *P* < 0.0001; Fig. 4c, e). In reciprocal experiments, we predicted that irreversible blockade of elongation would mask the effects of compounds that reduce initiation as the majority of ribosomes would be trapped in the elongation phase of translation. Significant changes were absent between samples containing inhibitors of any type or our negative controls (vehicle and scramble SPOT-ON; Fig. 4b, d, f). We conclude that the Poly(A) SPOT-ON likely impairs initiation consistent with the known role of PABP in stimulating cap-dependent translation via eIF4G^40–43^.

### Translation in sensory neurons

**Fig. 5:**
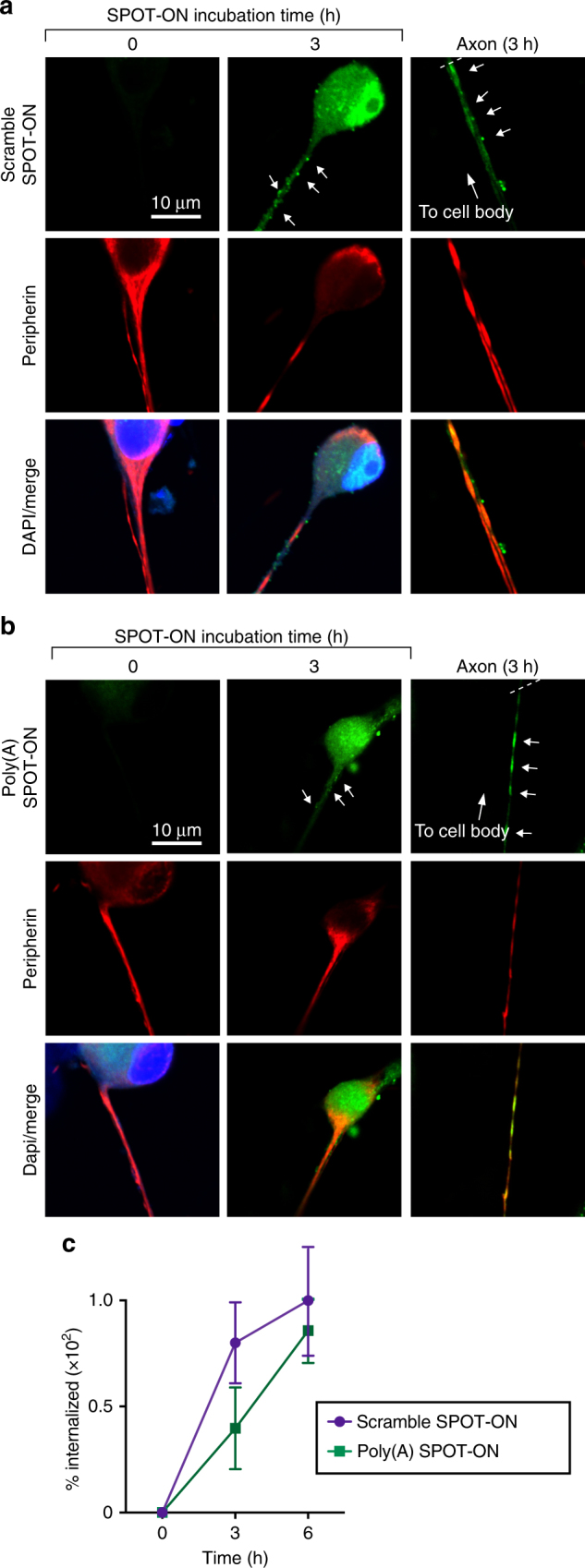
SPOT-ONs are taken up by cultured DRG sensory neurons. Uptake of SPOT-ONs was determined based on imaging of cultured DRG neurons over time. **a** Scramble SPOT-ON and **b** Poly(A) SPOT-ONs are taken up by DRG neurons and are localized into their axons after a 3-h period. **c** Quantification of SPOT-ONs uptake in DRG neurons from time zero to 6 h. *n* = 6. Data are plotted as mean ± s.e.m.

**Fig. 6:**
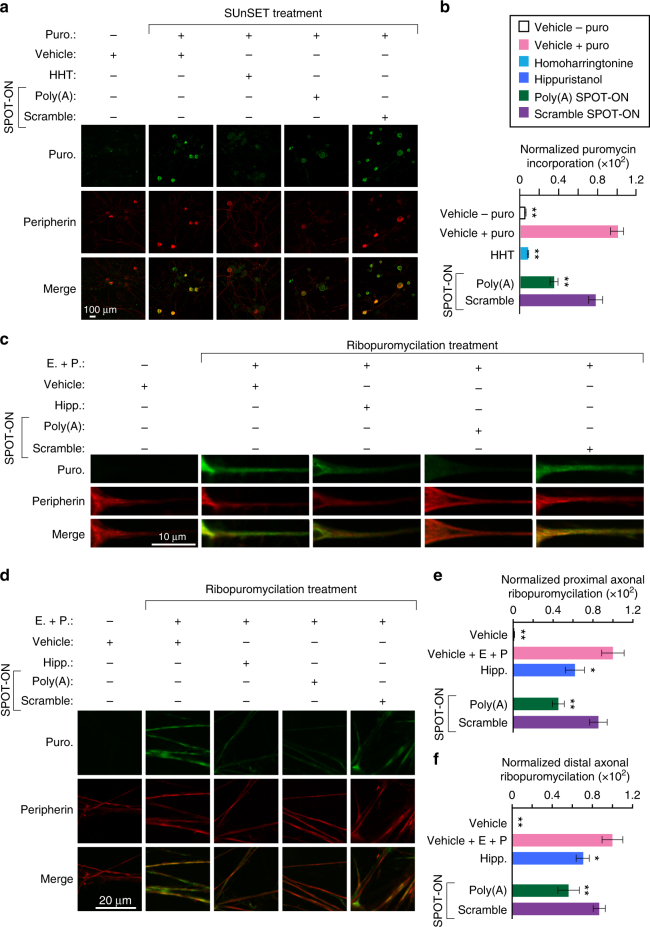
The Poly(A) SPOT-ON reduces nascent protein synthesis and axonal translation in DRG neurons. **a** Cultured DRG neurons are incubated with SPOT-ONs (10 μM) or homoharrintonine (50 μM) for 3 h prior to addition of puromycin (1 μM) for an additional 15 min. Incubation with Poly(A) SPOT-ON, but not scrambled SPOT-ON or vehicle, significantly reduces nascent protein synthesis in DRG neurons. Staining is shown from top to bottom for puromycin (green), peripherin (red), or a merge. **b** Quantification of **a**. *n* = 6. *P < 0.05, **P < 0.01, significantly different from vehicle + puro group analyzed by one-way ANOVA followed by Bonferroni post hoc test. **c** Cultured DRG neurons are incubated with vehicle, SPOT-ONs, or hippuristanol for 3 h followed by emetine incubation (200 μM) for 5 min and puromycin (100 μM) for an additional 5 min. Incubation with Poly(A) SPOT-ON (10 μM), but not scrambled SPOT-ON or vehicle, significantly reduces proximal axonal translation (around 20–25 μM from the cell body) in peripherin-positive DRG axons. As in **a**, staining is shown from top to bottom for puromycin (green), peripherin (red), or a merge. **d** Representative images showing distal axonal ribopuromycylation (more than 25 μM from the cell body; randomly selected) in peripherin-positive DRG axons under identical conditions as described in **c**. **e** Quantification of images in **c**. *n* = 20. *P < 0.05, **P < 0.01, significantly different from vehicle+E+P group analyzed by one-way ANOVA followed by Bonferroni post hoc test. **f** Quantification of images in **d**. *n* = 9. *P < 0.05, **P < 0.01, significantly different from vehicle +E + P group analyzed by one-way ANOVA followed by Bonferroni post hoc test. For all graphs shown in the figure, data are plotted as mean ± s.e.m.

First, we demonstrated that the SPOT-ONs are efficiently taken up and are distributed throughout the soma of DRG neurons including localization into their axons after a 3-h period (Fig. 5a–c). Second, to probe if sensory neurons responded to PABP inhibition in a similar way to cell lines, we determined rates of nascent protein synthesis in mouse DRG sensory neurons using SUnSET (Fig. 6a, b). To specifically mark neurons that are likely nociceptors, we scored only peripherin-positive cells. Robust translation was observed in the presence of puromycin (vehicle). Addition of either the general protein synthesis inhibitor homoharrintonine or the Poly(A) SPOT-ON significantly reduced protein synthesis (F_4, 26_ = 13.47, *P* < 0.0001; Fig. 6b). The scramble SPOT-ON failed to produce a significant effect. These results argue that the inhibitory effects of the SPOT-ONs are consistent between primary mouse neurons and our immortalized cell line.

Localized translation is fundamental to neuronal plasticity and has been linked to pain plasticity^44^. To ascertain if the SPOT-ON impairs axonal translation, we again utilized RPM to quantify protein synthesis levels in axons either proximal to the cell body or at distal regions in the presence or absence of the Poly(A) SPOT-ON (Fig. 6c, f). DRG neurons were treated with either hippuristanol, the Poly(A) SPOT-ON, or the scramble SPOT-ON. We found that addition of either hippuristanol or the Poly(A) SPOT-ON, but not scramble SPOT-ON or vehicle, significantly reduced proximal (*F*_4, 93_ = 10.63, *P* < 0.0001; Fig. 6c, e) and distal (*F*_4, 39_ = 19.34, *P* < 0.0001; Fig. 6d, f) axonal RPM staining in DRG sensory neurons. The RPM signal originating from distal axons is more diffuse than the punctate signal observed in dendrites from primary rat hippocampal neurons^39^. This may reflect experimental differences such as bona fide organizational changes in subcellular distribution of ribosomes. Our results from nociceptor axons are consistent with prior work suggesting that ribosomes in myelinated axons of lumbar spinal nerve roots are arranged in periaxoplasmic plaques^45^.

### PABP distribution in the peripheral nervous system

**Fig. 7:**
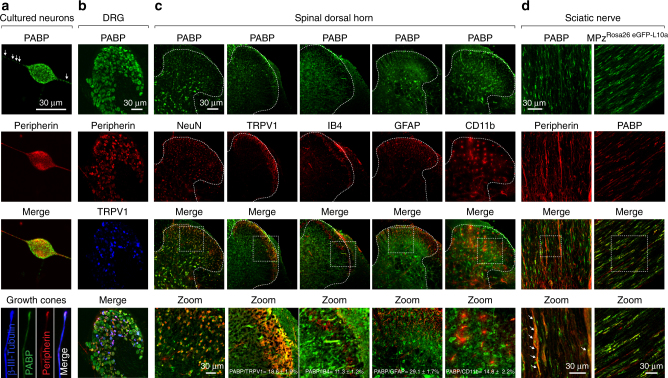
Binding protein PABP is present throughout the peripheral nervous system. **a** PABP (green) is highly expressed in cultured DRG neurons and their axons including growth cones and co-localizes with peripherin immunoreactivity, a marker for unmyelinated sensory neurons (red and merge). **b** PABP is broadly expressed in the majority of DRG neurons and co-localizes with peripherin and TRPV1, a nociceptive marker for both C and Aδ fibers. **c** PABP co-localizes with the neuronal marker NeuN and is also expressed in TRPV1-positive and IB4-positive pre-synaptic endings of DRG neurons in the spinal dorsal horn. PABP is also differentially expressed in microglia (CD11b+) and astrocytes (GFAP+) in the spinal dorsal horn. As shown in the figure, 18.6 ± 1.9% of the PABP immunoreactive fibers co-localize with TRPV1, 11.3 ± 1.2% with IB4, 29.1 ± 1.7% with GFAP, and 14.8 ± 2.2% with CD11b. *n* = 5 slices from L4–L6 spinal dorsal horn. Data are expressed as mean ± s.e.m. **d** PABP present in small-diameter sensory axons containing peripherin and in Schwann cells (MPz+) in the sciatic nerve

To characterize the cellular distribution of PABP in the primary nociceptive system, we used immunohistochemistry (Fig. 7). PABP was expressed in the soma of cultured DRG sensory neurons with high levels of PABP localizing into the axons (Fig. 7a). Next, the DRG (Fig. 7b), spinal dorsal horn (Fig. 7c), and sciatic nerve (Fig. 7d) were examined. Consistently with the expression in cultured DRG neurons, we found that PABP was broadly expressed and co-localized with peripherin immunoreactivity, a marker for unmyelinated, mostly nociceptive neurons. We also found that PABP was expressed in transient receptor potential cation channel subfamily V member 1 (TRPV1)-positive neurons in the DRG, indicating its presence in small-diameter, unmyelinated C-fibers and medium diameter, thinly myelinated Aδ fibers.

In the spinal cord dorsal horn, PABP is present in neurons as evidenced by co-localization with the neuronal marker NeuN. Moreover, PABP was differentially observed in isolectin B4 (IB4)-immunoreactive and TRPV1-immunoreactive fibers in the superficial layers of the dorsal horn. These results indicate that PABP is localized within pre-synaptic central terminals of nociceptive DRG neurons. Although PABP is present in nociceptive neurons suggesting a key role in axonal translation, it is also found in non-neuronal cells such as microglia and astrocytes in the spinal dorsal horn. Furthermore, PABP is located in the axons of the sciatic nerve in tissues as well as in cultured nociceptors. Consistent with the presence in non-neuronal cells in the spinal cord, PABP is also present in Schwann cells in the sciatic nerve as revealed by the co-localization with the myelin protein zero (MPz) protein. Together, this suggests that PABP might serve critical but unexplored roles in nociception, including regulating translation at the distal ends of nociceptors in the periphery and spinal dorsal horn.

### Inhibition of NGF- and IL-6-mediated allodynia

**Fig. 8:**
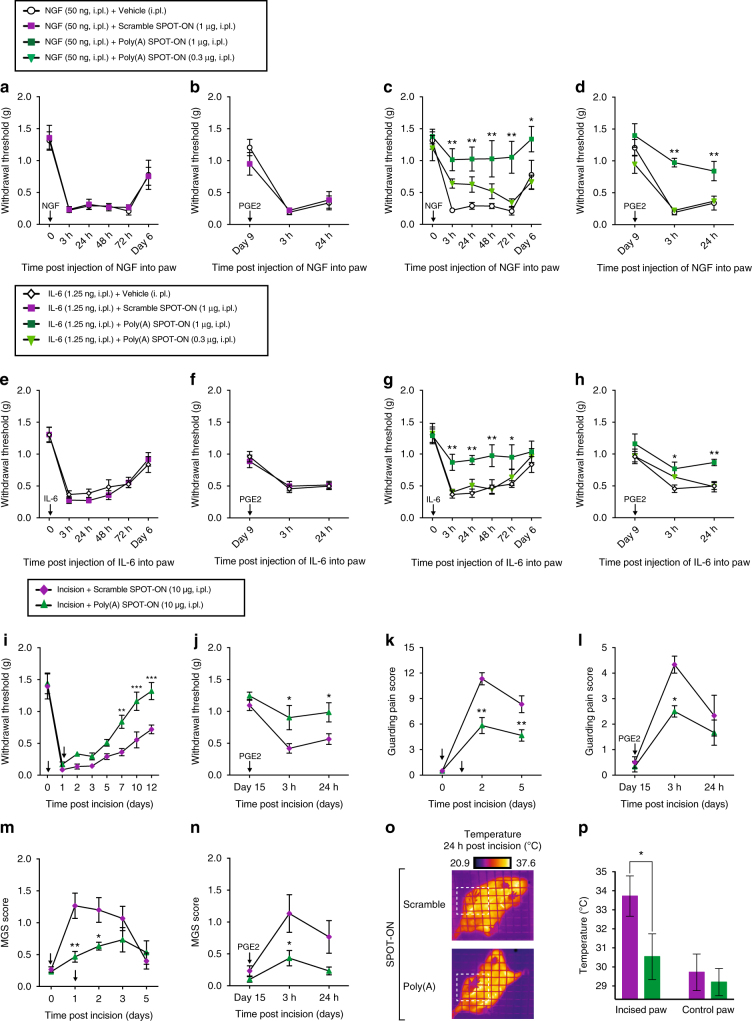
The Poly(A) SPOT-ON reduces pain sensitization in mice produced by intraplantar NGF or IL-6 administration and after plantar incision. **a**, **b** Intraplantar injection with vehicle or scrambled SPOT-ON (0.3–1 μg) did not reduce NGF-induced mechanical hypersensitivity or priming produced by intraplantar injection with PGE_2_ (100 ng) at day 9 after surgery. **c**, **d** Intraplantar injection with Poly(A) SPOT-ON (1 μg) reduces NGF-induced mechanical hypersensitivity and blocked the development of PGE_2_-induced hyperalgesic priming. *P < 0.05, **P < 0.01, significantly different from NGF+vehicle group analyzed by two-way ANOVA followed by Bonferroni post hoc test. **e**, **f** Intraplantar injection with vehicle or scrambled SPOT-ON (0.3–1 μg) did not reduce IL-6-induced mechanical hypersensitivity or priming produced by PGE_2_. **g**, **h** Intraplantar injection with Poly(A) SPOT-ON (1 μg) reduces IL-6-induced mechanical hypersensitivity and blocked the development of PGE_2_-induced hyperalgesic priming. *P < 0.05, **P < 0.01, significantly different from IL-6+vehicle group analyzed by two-way ANOVA followed by Bonferroni post hoc test. **i**, **j** Following plantar incision, local injection with Poly(A) SPOT-ON (10 μg), but not scrambled SPOT-ON (10 μg), reduces mechanical hypersensitivity, contributed to resolution of pain sensitization, and blocked development of hyperalgesic priming when animals were challenged with PGE_2_ at day 15. *P < 0.05, **P < 0.01, significantly different from incision+scramble group analyzed by two-way ANOVA followed by Bonferroni post hoc test. **k**, **l** Intraplantar injection of the Poly(A) SPOT-ON, but not scrambled SPOT-ON, significantly reduces the development of paw guarding following surgery as well as PGE_2_-induced priming. *P < 0.05, **P < 0.01, significantly different from incision+scramble group analyzed by two-way ANOVA followed by Bonferroni post hoc test. **m**, **n** Intraplantar injection of the Poly(A) SPOT-ON, but not scrambled SPOT-ON, significantly reduces the presence of facial grimace following surgery and after priming with PGE_2_. *P < 0.05, **P < 0.01, significantly different from incision+scramble group analyzed by two-way ANOVA followed by Bonferroni post hoc test. **o** Paw incision significantly increases the temperature in the incised paw of mice 24 h after surgery. Under these conditions, local administration of the Poly(A) SPOT-ON, but not scrambled SPOT-ON, significantly decreased the incised paw temperature 24 h after surgery. **p** Quantification of incised and non-incised paw temperature from scrambled and SPOT-ON groups 24 h after surgery. *P < 0.05, **P < 0.01, significantly different from incision+scramble group analyzed by Student's *t* test. *n* = 6 per group. For all graphs showing in the figure, data are plotted as mean ± s.e.m.

A standard method to evaluate allodynia in mice and humans is measuring mechanical sensitivity in response to von Frey filament application. Under normal conditions (no pain), plantar mechanical withdrawal threshold in mice is approximately 1.0–1.5 g force. However, after intraplantar injection of pro-inflammatory mediators or tissue injury, nociceptors become sensitive to mechanical stimulation. A drop or increase in the withdrawal threshold after insult is interpreted as hyperalgesia and analgesia, respectively. Commonly, NGF and IL-6 are used as pro-inflammatory mediators; both increase nociceptor excitability and induce plasticity, resulting in mechanical hypersensitivity^8^. After the resolution of the initial insult produced by NGF or IL-6, a long-lasting sensitivity to subsequent stimulation by the inflammatory mediator prostaglandin E2 (PGE_2_) is observed. PGE_2_ is commonly used as a mild stimulus that produces a short-term hypersensitivity in naïve animals. However, when animals are previously primed with noxious stimuli, PGE_2_ is now capable to produce a long-lasting hypersentivity. This event is referred to as hyperalgesic priming and is frequently associated with the process underlying the transition from acute to chronic pain^46^. We examined if the Poly(A) SPOT-ON impairs NGF-induced or IL-6-induced changes in mechanical hypersensitivity in vivo. We also assessed the presence of hyperalgesic priming in all groups 9 days after NGF or IL-6 treatment, a time point where animals had completely returned to baseline mechanical thresholds, by giving an intraplantar injection of PGE_2_. We did not observe any changes in NGF-induced mechanical hypersensitivity in the presence of vehicle or scramble SPOT-ON (Fig. 8a) or after precipitation of priming with PGE_2_ (Fig. 8b). However, the highest dose of Poly(A) SPOT-ON markedly inhibited NGF-induced mechanical hypersensitivity (*F*_2, 90_ = 26.59, *P* < 0.0001; Fig. 8c) and blocked the development of hyperalgesic priming (*F*_2, 45_  =  22.14, *P* < 0.0001; Fig. 8d). Likewise, IL-6-induced mechanical hypersensitivity and priming was not affected by vehicle or scramble SPOT-ON administration (Fig. 8e, f), but the Poly(A) SPOT-ON efficiently reduced mechanical hypersensitivity (*F*_2, 72 = _15.13, *P* < 0.0001; Fig. 8g) and the development of hyperalgesic priming (*F*_2, 42 = _9.935, *P* = 0.0003; Fig. 8h). These results suggest that the Poly(A) SPOT-ON blocks produce pain sensitization driven by NGF and IL-6 and the development of hyperalgesic priming.

### Incision-evoked pain responses

Both NGF and IL-6 are locally produced following tissue injury, including incision for surgery, where they are involved in producing prolonged hyperexcitability that promotes peripheral sensitization in nociceptors that innervate the injured area^47,48^. We tested whether the Poly(A) SPOT-ON would also inhibit incision-evoked pain in mice. We again assessed the presence of hyperalgesic priming in all groups 15 days after surgery when the animals had returned to baseline mechanical thresholds. Local injection at the time of incision and injection at the incision site 24 h after surgery with the Poly(A) SPOT-ON, but not scramble SPOT-ON, decreased incision-evoked mechanical hypersensitivity and contributed to the more rapid resolution of mechanical pain sensitization (*F*_1, 80 = _37.44, *P* < 0.0001; Fig. 8i). Injection of the Poly(A) SPOT-ON also blocked the development of hyperalgesic priming produced by incision (*F*_1, 30 = _13.57, *P* = 0.0009; Fig. 8j). In the same animals, we tested whether the Poly(A) SPOT-ON had an effect on incision-induced spontaneous pain responses. No paw guarding behavior was observed before plantar incision. However, robust paw guarding behavior was present in the incised paw following surgery and after demonstration of priming with PGE_2_ (Fig. 8, l). Local injection of the Poly(A) SPOT-ON, but not the scramble SPOT-ON, significantly reduced the development of paw guarding following surgery (*F*_1, 30 = _28.7; *P* < 0.0001; Fig. 8k) as well as when the animals were subsequently challenged with PGE_2_ 15 days after incision (*F*_1, 30 = _6.214, *P* = 0.0184; Fig. 8l). Using the same protocol, we recorded the affective component of pain by scoring the facial expressions of the animals before and after surgery based on facial cues. In this model, an increase in the Mouse Grimace Scale (MGS) was observed following surgery and after demonstration of priming with PGE_2_ (Fig. 8m, n). Local injection of the Poly(A) SPOT-ON, but not the scramble SPOT-ON, significantly reduced the development of facial grimace following surgery (*F*_1, 50 = _12.03, *P* = 0.0011; Fig. 8m) and 3 h after hyperalgesic priming revealed by PGE_2_ injection (*F*_1, 30 = _10.44, *P* = 0.0030; Fig. 8n). Finally, we determine the thermal changes in the incised vs. non-incised paw as an indirect measure of inflammation using a similar approach previously reported for inflammatory and arthritic pain models^49^. We reasoned that pro-inflammatory mediators released at the site of surgery produce inflammation and, at the same time, an increase in paw temperature due to enhanced blood flow. Incised paws displayed increased temperature 24 h after surgery (Fig. 8o, p). We did not observe any thermal changes in the non-incised paw after surgery, indicating that pro-inflammatory mediators are released only in the inflamed area. Local administration of the Poly(A) SPOT-ON, but not the scramble SPOT-ON, significantly decreased the incised paw temperature when mice were assessed 24 h after surgery (*t *= 2.795, *P* = 0.0209; Fig. 8o, p). Taken together, our results indicate that local treatment with the Poly(A) SPOT-ON can be a potentially efficacious treatment for the prevention of pain and inflammation brought about by tissue injury.

### Capsaicin-induced inflammatory pain

**Fig. 9:**
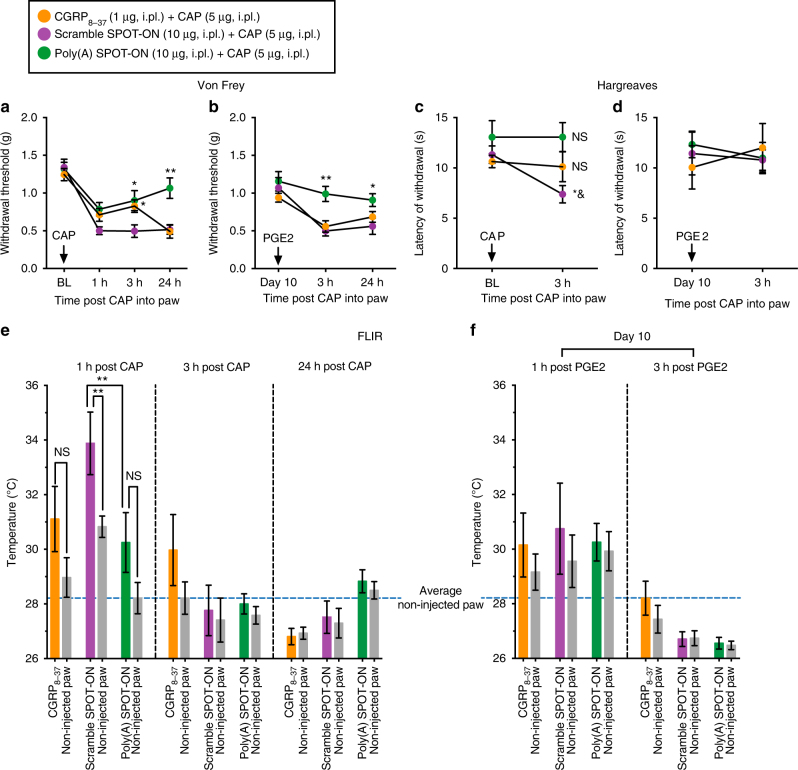
The Poly(A) SPOT-ON reduces pain sensitization produced by capsaicin. **a** The Poly(A) SPOT-ON (10 μg) inhibits the mechanical hypersensitivity produced by intraplantar capsaicin (5 μg) and **b** blocks the development of hyperalgesic priming. CGRP_8–37_ (1 μg) has a transient antinociceptive effect at 3 h post capsaicin with no changes after the precipitation of priming with PGE_2_. *P < 0.05, **P < 0.01, significantly different from scramble SPOT-ON+capsaicin (CAP) group analyzed by two-way ANOVA followed by Bonferroni post hoc test. **c** The Poly(A) SPOT-ON and CGRP_8–37_ attenuate the thermal hypersensitivity produced by capsaicin. *P < 0.05, significantly different from Poly(A) SPOT-ON+capsaicin (CAP) group and ^&^ *P* < 0.05, significantly different from baseline (BL) analyzed by two-way ANOVA followed by Bonferroni post hoc test. Not significantly different (NS) compared to baseline (BL). **d** No changes in thermal hypersensitivity are detected after priming revealed by PGE_2_. **e** The Poly(A) SPOT-ON and CGRP_8–37_ block the transient increase in paw temperature produced by intraplantar capsaicin administration. **P < 0.01, significantly different from the non-injected paw or the Poly(A) SPOT-ON injected paw analyzed by one-way ANOVA followed by Bonferroni post hoc test. Not significantly different (NS) compared to non-injected paw. **f** No changes in paw temperature are present after priming (injected vs. non-injected paw). *n* = 6 per group. For all graphs shown in the figure, data are plotted as mean ± s.e.m.

Neurogenic inflammation plays a key role in nociceptor sensitization by a mechanism that is partially driven by the neuropeptide release, such as CGRP, from primary afferent fibers in response to noxious stimuli including capsaicin, an agonist of TRPV1 channels^50^. In order to show more evidence that nociceptors are relevant targets of the Poly(A) SPOT-ON, we used capsaicin as an inflammatory mediator because of its very specific interaction with nociceptors. This idea was justified based on the results showing the presence of PABP in TRPV1-positive neurons in the DRG and pre-synaptic endings in the spinal dorsal horn (Fig. 7b, c). Intraplantar injection of capsaicin produced mechanical and thermal hypersensitivity together with a transient increase in paw temperature (Fig. 9). The Poly(A) SPOT-ON, but not the scramble SPOT-ON, inhibited capsaicin-induced mechanical hypersensitivity (*F*_2, 56 = _11.06, *P* < 0.0001; Fig. 9a) and blocked the development of hyperalgesic priming (*F*_2, 45_ = 9.801, *P* = 0.0003; Fig. 9b). Moreover, CGRP_8–37_, a CGRP receptor antagonist, had a transient antinociceptive effect at 3 h post capsaicin administration and did not block the precipitation of hyperalgesic priming at day 10 (Fig. 9a, b). Similarly, development of thermal hypersensitivity was attenuated by the Poly(A) SPOT-ON and CGRP_8–37_ with no significant antinociceptive effects observed in the scramble SPOT-ON group (*F*_2, 30 = _4.972, *P* = 0.0137; Fig. 9c). However, no changes in thermal hypersensitivity were detected in any groups after priming revealed by PGE_2_ injection (Fig. 9d). Coupling thermal hypersensitivity with forward looking infrared (FLIR) imaging, we observed that the Poly(A) SPOT-ON and CGRP_8–37_, but not the scramble SPOT-ON, blocked the transient increase in paw temperature produced by intraplantar capsaicin administration (*F*_5, 30 = _4.741, *P* = 0.0026; Fig. 9e). Similar to the thermal hypersensitivity data, no changes in capsaicin-injected paws compared to non-injected paws were detected with FLIR after priming revealed by PGE_2_ (Fig. 9f). Together, these results demonstrate that part of the effect produced by the Poly(A) SPOT-ON is mediated by blocking induction of axonal plasticity in primary afferent fibers responsive to capsaicin.

## Discussion

Our experiments permit four major conclusions. First, RNA-based SPOT-ON “decoys” can inhibit RNA–protein interactions and are functional in vivo. Second, PABPs are broadly distributed in the nociceptive pathway and play critical roles in protein synthesis. Third, inhibition of PABPs with SPOT-ONs can robustly impair pain behavior. Fourth and finally, PABP inhibition diminishes inflammation following incision or intraplantar capsaicin administration.

We determined the sequence preferences of a conserved translation factor and applied this information toward the generation of a competitive inhibitor RNA. This constitutes, to the best of our knowledge, the first such attempt to disrupt RNA–protein interactions through the use of chemically stabilized mimetics. This approach is particularly well suited to PABPs given their essential requirement in basal eukaryotes such as yeast and in animals. SPOT-ONs are rapidly taken up by cells and lack overt signs of toxicity. The SPOT-ONs we report are not tailored for uptake by a specific cell type and could be improved upon through targeting moieties for nociceptor neurons. Similar approaches devised to improve delivery of microRNA antagonists could in principle improve the potency of SPOT-ONs in vivo^51^.

The implications of our approach are broad given the function of the Poly(A) SPOT-ON in vivo and the need to understand the function of the more than 800 RNA-binding proteins found in the human genome^52^. The modifications introduced into the SPOT-ON were well tolerated by PABP; the Poly(A) SPOT-ON binds with comparable affinity to an unmodified substrate and appears to be highly specific in gel-shift and cell-based measurements. PABP is abundant in the cell and has a moderate affinity for Poly(A) RNA. Our ability to competitively inhibit its function bodes well for RNA-binding proteins as a class given that many recognize more complex elements with higher affinity. This approach may broadly provide a means to interrogate the function of RNA-binding proteins whose specificity is distinct through the use of similar RNA-derived decoys.

PABPs are present in the peripheral nervous system. While abundant in the somas of DRG neurons, they are also clearly present in axons. This contributes to a growing body of evidence in support of PABP as an active participant in RNA localization and in localized translation. For instance, PABP is present in dendrites and terminal growth cones and binds to localized regulatory RNAs including BC1 and BC200^53,54^. PABP physically associates with proteins that modulate local protein synthesis in dendrites such as Makorin RING (Really Interesting New Gene) zinc-finger protein-1^55^. Finally, PABP is present in neuronal granules containing proteins implicated in activity-dependent protein synthesis and RNA localization including: HuD Staufen, Zip-code-binding protein, and Pumilio^56,57^. Our results indicate that the Poly(A) SPOT-ON reduces nascent protein synthesis in both axons and cell bodies in vitro. This raises the question as to which site is relevant for the behavioral effects of PABP inhibition. As the site of delivery was the paw where axons reside, distal to cell bodies located in ganglia, one potential mechanism for the effects of the Poly(A) SPOT-ON is in axons. However, the expression of PABP in non-neuronal cells near the site of injection, including resident immune cells, underscores the ubiquitous distribution of PABP. The Poly(A) SPOT-ON is not specifically targeted to neurons and appears to be readily taken up by other cell types. Thus, we cannot exclude the possibility that non-neuronal mechanisms contribute to the observed series of pharmacological effects. Our experiments contribute additional understanding to the potential biological roles of PABP in nociception. Genetic loss of PAIP suggests that exaggerated PABP activity has no apparent consequence on mechanical sensitivity^58^. We observed that the Poly(A) SPOT-ON elicits substantial antihyperalgesic effects on mechanical hypersensitivity. Additional experiments are required to examine the downstream targets of the Poly(A) SPOT-ON.

Sensory neurons are key mediators of nociceptive sensitization. In the peripheral nociceptive system, local protein synthesis in nociceptor terminals or their distal axons has been implicated in promoting hyperexcitability and producing pain sensitization^59^. Inhibition of activity-dependent translation in axons blocks the development of persistent plasticity as measured by the presence of hyperalgesic priming. This strongly suggests that development of chronic pain requires regulated local protein synthesis. Thus, understanding basic mechanisms that drive pain sensitization is crucial for the identification of potential targets for chronic pain treatment. Our data indicate that PABP inhibition can impact behavioral plasticity after injury. This contributes to prior work on the 3′ end in nociceptive sensitization. For instance, local administration of an inhibitor of mRNA polyadenylation (cordyceptin) prevents hyperalgesic priming in rats^60^. Additionally, the cytoplasmic polyadenylation element-binding (CPEB) RNA-binding protein contributes to nociceptor plasticity^61^. CPEB is a target of calmodulin-activated protein kinase IIα and mediates regulated cytoplasmic polyadenylation. Taken together, these experiments support a model wherein dynamic extension of the Poly(A) tail facilitates nociceptor axonal plasticity. The inclusion of PABP in this model provides a vital link between the 3′ end of mRNA and factors bound to the m^7^G cap via eIF4G.

In our in vivo experiments, we noted a decrease in pain induced by incision using mechanical stimulation, paw guarding, and facial grimace assessment. Similarly, surgically induced inflammation or capsaicin-induced inflammatory pain were decreased as a result of treatment with the Poly(A) SPOT-ON. Is the Poly(A) SPOT-ON targeting neurons or immune cells to reduce pain and inflammation? We favor a scenario where the Poly(A) SPOT-ON preferentially targets nociceptors to reduce pain and inflammation. Guarding and grimace behavior is induced by ongoing nociceptor activity after injury. This ongoing activity also drives neurogenic inflammation which is a critical contributor to inflammation after injury. Neurogenic inflammation is primarily driven by CGRP release from nociceptors that trigger blood vessels to promote blood flow to the injured area^50^. The capsaicin data suggest that the Poly(A) SPOT-ON blocks plasticity by a factor that has a very specific interaction with nociceptors. The combination of behavioral results using evoked and non-evoked stimuli, when taken together with the temperature-based inflammation measures, is consistent with a neuronally mediated mechanism of action for the Poly(A) SPOT-ON. However, our data do not exclude the possibility of a contribution from non-neuronal cell types.

To summarize, numerous mechanisms govern plasticity in nociceptors. Several of these mechanisms converge on regulated changes in Poly(A) tail length. Through the use of chemically modified RNAs, we provide evidence that PABP plays an integral function in signal integration in response to inflammatory models of pain in mice. Our approach suggests that targeting RNA–protein interactions may provide a new source of pharmacological agents for probing mechanism of action in vivo. This is a particularly important question given the preponderance of RNA-binding proteins encoded by the human genome whose associations with RNA lack overt function^52^. The use of the SPOT-ON approach provides a novel means to interrogate this problem.

## Methods

### In vitro selection and high-throughput sequencing and sequence specificity landscapes (SEQRS)

SEQRS was conducted as described with minor modifications on *PABPC1* ^62^. The initial RNA library was generated from transcription of 1 μg of double-stranded DNA using the AmpliScribe T7-Flash Transcription Kit (Epicentre). DNA was removed through incubation with Turbo DNAse. Two hundred nangorams of RNA was added to 100 nM *PABPC1* immobilized onto magnetic glutathione *S*-transferase (GST) resin (Fisher). Binding reactions were conducted in 100 μL of SEQRS buffer—50 mM HEPES, pH 7.4, 2 mM ethylenediaminetetraacetic acid (EDTA), 150 mM NaCl, 0.1% NP40, 1 mM dithiothreitol (DTT), 200 ng yeast transfer RNA (tRNA) competitor, and 0.1 U of RNase inhibitor (Promega). Magnesium and other metals catalyze non-specific cleavages in RNA; thus, a small amount of EDTA was included to enhance RNA integrity. An additional implication of EDTA in SEQRS is reduced preservation of structured RNAs throughout selection. Samples were incubated for 30 min at 22 °C prior to magnetic isolation of protein–RNA complexes. Unbound RNAs were aspirated and the beads were subjected to four washes with 200 μL of SEQRS buffer. After the final wash step, resin was suspended in elution buffer (1 mM Tris, pH 8.0) containing 10 pmol of the reverse transcription primer. Samples were heated to 65 °C for 10 min and then cooled on ice. Reverse transcription was conducted with ImProm-II reverse transcription reaction (Promega). The ssDNA product was used as a template for 25 cycles of PCR using a 50 μL GoTaq reaction (Promega). Sequencing data were processed as described^62^. Sequence logos corresponding to consensus binding motifs were generated by weblogo from the top 300 most enriched sequences. To calculate the area under the curve, two likelihood distributions were used. The data were partitioned into test and training sets. The training sets were used to learn the data likelihood function. Using the learned likelihoods and the test dataset, the ROC was formed for each fold. Finally, the ROCs were averaged over the 10-folds. The total area under the curve was calculated based on a trapezoidal approximation. Frequency distributions of SEQRS sequences in CLIP data were determined based on histograms of cumulative distributions surrounding sites of productive crosslinking across the genome as described^23^.

### Cell cultures

U2OS cell line cultures: The U2OS human osteosarcoma cell line was a gift from Dr. Shigeki Miyamoto (UW-Madison). Cells were cultured in high glucose Dulbecco’s modified Eagle’s Medium (DMEM, Corning) supplemented with 10% fetal bovine serum (FBS; Atlanta Biologicals). Cells were maintained at 37 °C in a humidified incubator with 5% CO_2_.

DRG neuronal cell cultures: Tale Swiss Webster mice (Taconic laboratories, 15–25 g) were used. DRGs from all levels were excised aseptically and placed in Hanks' balanced salt solution (HBSS; Invitrogen) on ice. The ganglia were dissociated enzymatically with collagenase A (1 mg/mL, 25 min, Roche) and collagenase D (1 mg/mL, Roche) with papain (30 U/mL, Roche) for 20 min at 37 °C. DRGs were then triturated in a 1:1 mixture of 1 mg/mL trypsin inhibitor (Roche) and bovine serum albumin (BioPharm Laboratories), then filtered through a 70 μm cell strainer (Corning). Cells were pelleted, then resuspended in DMEM/F12 with GlutaMAX (Thermo Fisher Scientific) containing 10% (Thermo Fisher Scientific), 1% penicillin and streptomycin, and 3 μg/mL 5-fluorouridine with 7 μg/mL uridine to inhibit mitosis of non-neuronal cells and were distributed evenly in poly-d-lysine-coated coverslips (BD Falcon) and incubated at 37 °C in a humidified 95% air/5% CO_2_ incubator for 6 days.

### Electrophoretic mobility shift

U2OS cell protein extracts of approximately 10 mg/mL were prepared with Ambion PARIS Kit (Thermo Fisher Scientific) according to the manufacturer’s instructions. In brief, cells were washed once with cold phosphate-buffered saline (PBS), lysed in cell fractionation buffer, and incubated on ice for 10 min. Cytoplasmic lysate was collected after centrifugation for 5 min at 500 × *g*. One microliter of 100 μM cyanine 3 phosphoramidite (Cy3)-labeled SPOT-ON was mixed with different amounts of protein lysate (0, 1, 2, 3, and 4 μL) in electrophoretic mobility shift assay (EMSA) buffer (10 mM HEPES, pH 7.4; 50 mM NaCl; 1 mM EDTA; 0.1 mg/mL bovine serum albumin; 0.01% (v/v) Tween-20, and 0.1 mg/mL yeast tRNA) and incubated on ice for 90 min. Three microliters of loading dye (15% (v/v) Ficoll 400 and 0.01% (v/v) bromophenol blue) was added to each 15 μL reaction before loading on the 6% DNA retardation gel (Invitrogen) in 0.5X TBE buffer at 100 V at 4 °C for 90 min. The gel was imaged with a Typhoon FLA7000 scanner (GE Healthcare).

### RNA immunoprecipitation

U2OS cells were washed once with cold PBS and lysed in cold TNMEN-150 buffer (50 mM Tris, pH 8; 1 mM EDTA; 2 mM MgCl_2_; 150 mM NaCl and 0.5% (v/v) NP40) with 100 U/mL RNase inhibitor (Promega) and protease inhibitor (Roche). The cells were incubated on ice for 30 min, then centrifuged at a maximum speed for 10 min at 4 °C. To generate PABP-depleted extracts, GST-tagged PAIP was purified in lysis buffer (50 mM Tris, pH 8; 500 mM NaCl; 0.1% (v/v) NP40; 1 mM MgCl_2_; 1% glycerol; 5 mM DTT and supplemented with protease inhibitor). The protein lysate was incubated with glutathione agarose resin (Gold Biotechnology). One hundred microliters of aliquots of U2OS lysate was incubated at 4 °C for 1 h with GST-tagged PAIP which was already immobilized with glutathione agarose resin. Equal amounts of U2OS lysate was incubated with resin alone as a mock control. The lysate–resin mixture was centrifuged at 500 rpm for 5 min at 4 °C. Supernatant was collected for the EMSA and RNA immunoprecipitation experiments. After PABP depletion, the supernatant was transferred to a new tube containing 15 μL of 100 μM biotin-labeled SPOT-ON and incubated on ice for 40 min. Twenty-five microliters of pre-equilibrated magnetic streptavidin beads (Pierce) were added to the SPOT-ON–biotin–lysate mixture and incubated for 80 min at 4 °C with continuous end-over-end rotation. Samples were then placed on a 96-well magnetic block and the beads were washed six times with cold wash buffer (50 mM Tris, pH 8; 1 mM EDTA; 2 mM MgCl_2_; 150 mM NaCl and 0.05% (v/v) NP40). After the final wash step, beads were resuspended in 25 μL of 1× sodium dodecyl sulfate-polyacrylamide gel electrophoresis (SDS-PAGE) loading buffer and boiled for 5 min. Electrophoresis was conducted on 4–15% gradient SDS-PAGE gels (Bio-Rad) before transferring to nitrocellulose membrane. The membrane was probed with PABP antibody (1:500; Santa Cruz, sc-32318) followed by horseradish peroxidase‐conjugated goat anti-mouse secondary antibody (1:300; Thermo Fisher Scientific, 32430). The signal was detected using ECL Select chemiluminescent substrate (GE Healthcare) on ChemiDoc Touch Imaging System (Bio-Rad).

### Florescence polarization

Equilibrium dissociation constants were determined by florescence anisotropy measurements of either unmodified adenosine 12 nucleotide RNA or the Poly(A) SPOT-ON to recombinant human *PABPC1* (residues 1–383). Binding reactions were conducted in 50 μL of buffer containing 50 mM HEPES, 5 mM EDTA, 250 mM KCl, 10 mM DTT, 0.5 mg/mL BSA, 0.05% Tween-20, 0.1 mg/mL yeast competitor total RNA (Ambion), and 0.5 nM Cy3-labeled RNA. Measurements were recorded on a Tecan Spark multimode plate reader in triplicate. Data were fit using Kalidagraph as described^63^.

### SPOT-ON stability

For U2OS cells, Cy3 3′-labeled SPOT-ONs were added respectively to DMEM media supplemented with 10% FBS and incubated at 37 °C at different time points (0, 0.5, 1, 2, 3, 6, and 24 h; unmodified Poly(A)_12_-Cy3 samples at 0, 12, 24, 36, and 48 h) were run on 6% DNA retardation gel in 0.5X TBE buffer. The gel was imaged with a Typhoon FLA7000 scanner (GE Healthcare).

### SPOT-ON uptake

For U2OS cells, Cy3 3′-labeled SPOT-ONs were added to DMEM media supplemented with 10% FBS and incubated at 37 °C at different time points (0, 0.5, 1, 2, 3, 6, and 24 h). For DRG neurons, wells were incubated with Cy3 3′-labeled SPOT-ONs for 3 and 6 h. After SPOT-ON incubation, samples were processed for immunofluorescence.

### Transient transfection

U2OS cells at 60–70% confluence were transfected with 0.5g, 1, and 2 μg of pcDNA3.1-PABP or pcDNA3.1 empty vector, respectively, using Lipofectamine 3000 (Invitrogen) according to the manufacturer’s instructions for 48 h. The cells were lysed and protein was extracted by ultrasonication in lysis buffer (50 mM Tris, pH 7.4, 150 mM NaCl, 1 mM EDTA, pH 8.0, and 1% Triton X-100) containing protease and phosphatase inhibitors (Sigma-Aldrich). Clear lysate was collected by centrifugation at 14,000 × *g* for 20 min at 4 °C. Protein samples in 1× Laemmli sample buffer (Sigma) was loaded and separated by 10% SDS-PAGE gels before transferring to Immobilon-P membranes (Millipore). The membrane was blocked in 5% milk for 1 h at room temperature, then incubated with PABP antibody (1:1000; cat. # ABE40, Millipore) overnight at 4 °C followed by goat anti-rabbit antibody conjugated to horseradish peroxidase (1:10,000; cat. # 111-036-144, Jackson ImmunoResearch). The signal was detected using Pierce ECL Western Blotting Substrate (Thermo Fisher) on ChemiDoc Touch Imaging System (Bio-Rad). The blot was stripped in Restore Plus western blot stripping buffer (Thermo Fisher) according to the manufacturer’s instructions and re-probed with c-Myc antibody (1:1000; cat. # MA1-980, Thermo Fisher) overnight followed by goat anti-mouse antibody conjugated to horseradish peroxidase (1:10,000; cat. # 115-035-174, Jackson ImmunoResearch). After the signal was detected, the blot was stripped again and re-probed with glyceraldehyde 3-phosphate dehydrogenase (GAPDH) antibody (1:10,000; cat. # 2118S, Cell Signaling) and goat anti-rabbit secondary antibody for GAPDH expression detection.

### SUnSET and RPM assays

In the SUnSET assay^34^, DRG neurons were cultured for 6 days in vitro. U2OS cells were plated on slides the day before the experiment to reach 70% confluence at the time of treatment. Test compounds (SPOT-ONs (10 μM) or homoharringtonine (50 μM)) were allowed to incubate for 37 °C for 3 h prior to the addition of puromycin (1 μM) for an additional 15 min. Immediately following the puromycin incubation, cells were washed in chilled HBSS containing 0.00036% digitonin (Sigma) for 2 min prior to fixation for the removal of background puromycin. In the RPM assay^39^, cultures and treatments were conducted in an identical way to the aforementioned SUnSET assay. However, after incubation of test compounds, emetine (200 μM) was then added for 5 min and puromycin (100 μM) was added for an additional 5 min. Cells were washed with cold 0.00036% (v/v) digitonin prior to immunofluorescence.

### Immunofluorescence

U2OS cell line cultures: Cells were fixed in 2% (v/v) formaldehyde (Thermo Fisher Scientific) in wash buffer (1% (v/v) BSA in PBS) at room temperature for 20 min. After washing three times with wash buffer, cells were permeabilized with 0.05% (v/v) saponin (Calbiochem) for 15 min, washed three times, and blocked in 10% (v/v) immunopure goat serum (MP Biomedicals) for 1 h. After three more washes, cells were stained with puromycin antibody (1:5000; Millipore, MABE343) and phalloidin-tetramethylrhodamine antibody (1:200; Sigma, P1951) at 4 °C overnight followed by goat anti-mouse antibody conjugated to Cyanine 5 (1:2000; Molecular Probe, A10524) at room temperature for 1 h. After three washes, cells were stained with 4',6-diamidino-2-phenylindole (285 nM) for 15 min and mounted with Prolong Diamond antifade mountant (Thermo Fisher Scientific).

DRG neuronal cell cultures: Cells were fixed in ice-cold 10% formalin in 1× PBS for 1 h. Cells were then washed with 1× PBS and permeabilized in PBS containing 10% heat-inactivated normal goat serum (NGS, Atlanta Biologicals, Atlanta, GA, USA) and 0.02% Triton X-100 (Sigma) in 1× PBS for 30 min and then blocked in 10% NGS in PBS for at least 1 h. Following additional washes, primary antibodies were used to detect the following proteins: PABP1 (1:500; cat. # ABE40, Millipore), peripherin (1:1000; cat. # P5117 or cat. # SAB4502419, Sigma-Aldrich), and puromycin (1:5000; Millipore, MABE343). Primary antibodies were applied overnight at 4 °C and the next day appropriate secondary antibodies (Alexa Fluor, Invitrogen) were applied for 1 h. After additional PBS washes, coverslips were mounted on frosted slides with ProLong Gold antifade (Invitrogen).

In order to visualize the presence of PABP in growth cones, DRG neurons at day 4 in vitro were cultured, fixed, permeabilized, blocked, incubated, and mounted using similar conditions aforementioned. The presence of PABP in growth cones was identified using specific antibodies for β-III tubulin (1:1000, cat. # G712A, Promega), PABP1 (1:500; cat. # ABE40, Millipore), and peripherin (1:1000, cat. # CPCA-peri, EnCor Biotechnology).

Tissues: Mice were anesthetized with isoflurane and euthanized by decapitation and tissues were flash frozen in O.C.T. on dry ice. Spinal cords were pressure ejected using chilled 1× PBS. Sections of spinal cord (20 μm), DRG (20 μm), and sciatic nerve (20 μm) were mounted onto SuperFrost Plus slides (Thermo Fisher Scientific, Waltham, MA, USA) and fixed in ice-cold 10% formalin in 1× PBS for 1 h and then subsequently washed three times for 5 min each in 1× PBS. Slides were then transferred to a solution for permeabilization made of 1× PBS with 0.2% Triton X-100 (Sigma-Aldrich). After 30 min, slides were washed three times for 5 min each in 1× PBS. Tissues were blocked for at least 2 h in 1× PBS and 10% heat-inactivated NGS. Primary antibodies were used to detect the following proteins: PABP1 (1:500; cat. # ABE40, Millipore), PABP4 (1:500; cat. # A301-467, Bethyl Laboratories), NeuN (1:1000; cat. # MAB377, Millipore), peripherin (1:1000; cat. # P5117 or cat. # SAB4502419, Sigma-Aldrich), TRPV1 (1:1000; GP14100, Neuromics), CD11b (1:1000; cat. # T-3102, BMA Biomedicals), and GFAP (1:1000; cat. # sc-33673, Santa Cruz Biotechnology). Primary antibodies were applied and incubated with spinal cord, DRG, and sciatic nerve sections on slides at 4 °C overnight. The next day, appropriate secondary antibodies (Alexa Fluor, Invitrogen) were applied for 1 h. After additional 1× PBS washes, coverslips were mounted on frosted slides with ProLong Gold antifade (Invitrogen). Cells or tissues from all groups were processed together under identical conditions with the same reagents and confocal microscopy images were obtained with an Olympus FluoView 1200 single-photon confocal microscope.

### Image acquisition analysis

To calculate the puromycin incorporation and the distal ribopuromycylation, image analysis was performed using the ImageJ plug-in JACoP (Just Another Co-localization Plugin) (http://rsb.info.nih.gov/ij/plugins/track/jacop2.html)^64^. Manders’ overlap coefficient M1 (peripherin/puromycin; using thresholds) was calculated in images collected from all groups. The M1 coefficient will vary from 0 to 1, the former corresponding to non-overlapping images and the latter reflecting 100% co-localization between both images. The M1 overlap coefficient values obtained from all groups were normalized to vehicle+puromycin group values and expressed as % of normalized puromycin incorporation.

To calculate the ribopuromycylation in proximal axons, the corrected total cell fluorescence (CTCF) was used to quantify the intensity of the puromycin signal for individual axons between experimental groups. In order to do so, an outline was drawn around the axons starting near to the cell bodies and extended up to 25 μm away from them. Using ImageJ, the integrated density and the area, as well as the background noise was measured and the CTCF calculated as equal to the integrated density − (area of selected cell × mean fluorescence of background readings). CTCF values from all groups were normalized to vehicle+puromycin group values and expressed as % of normalized proximal RPM.

To determine PABP immunoreactivity in either TRPV1-positive, CGRP-positive, and IB4-positive fibers or CD11b-positive and GFAP-positive cells, intensity correlation analysis (ICA) was calculated for regions of interest (ROI) in images (*n* = 5 slices) collected from the L4–L6 region of the lumbar spinal dorsal horn. ICA computes the sum of (current pixel intensity in channel A − channel A’s mean intensity) ×  (current pixel intensity in channel B − channel B’s mean intensity) for each ROIs. Percentage of A channel over B channel intensity correlation is represented.

### NGF and IL-6 models of hyperalgesic priming

A mouse model for “hyperalgesic priming” originally developed by Levine and colleagues^65^ was used for the study. Animals were placed in acrylic boxes with wire mesh floors, and baseline plantar mechanical sensitivity was measured after habituation for 1 h using the up-down method^66^. Briefly, Von Frey monofilaments (Stoelting, Wood Dale, IL, USA) were firmly applied to the plantar surface of left hindpaw for 5 s and the up-down method was used to estimate the withdrawal threshold in grams (g). To establish hyperalgesic priming, we co-administered the SPOT-ONs (0.3, 1 μg) with recombinant mouse IL-6 (1.25 ng; R&D Systems) or mouse 2.5S NGF (50 ng; Millipore) in 25 μL sterile PBS into the left hindpaw with an intraplantar (i.pl.) injection and measured their mechanical withdrawal thresholds at various time points after administration. Following complete resolution of the initial mechanical hypersensitivity (day 9), mice were again assessed for their mechanical withdrawal threshold and subsequently injected into the left hindpaw with PGE_2_ (100 ng; Cayman Chemical) in 25 μL sterile 0.9% NaCl. Afterwards, mechanical withdrawal thresholds were measured at 3 and 24 h post PGE_2_.

### Plantar incision model

Prior to surgery all animals were assessed for baseline paw withdrawal thresholds using the up-down method. Baseline paw guarding, thermal, and grimace thresholds were assessed according to the methods described below. Plantar incision was performed as described previously^67^. A 5 mm longitudinal incision was made with a number 11 blade through skin, fascia, and muscle of the plantar aspect of the hindpaw in isoflurane-anesthetized mice. The skin was apposed with two sutures of 5 mm silk and immediately after mice received an intraplantar injection with SPOT-ONs in the incised paw and one more injection at 24 h in a total volume of 25 μL sterile PBS. Following complete resolution of the mechanical hypersensitivity (day 15), mice were administered PGE_2_ (100 ng; Cayman Chemical) into the plantar surface of the incised paw in a total volume of 25 μL.

MGS was used to quantify spontaneous pain in mice^68^. We scored the changes in the facial expressions at different time points after incision and after i.pl. PGE_2_ injection. In this method, all faces are to be coded for the presence and intensity of the following specific facial action units (AU): orbital tightening, nose bulge, cheek bulge, ear position, and whisker change. Intensity ratings are coded for each AU as follows: AU is not present = 0, AU moderately visible = 1, and AU severe = 2. An MGS score for each mouse is calculated by averaging intensity ratings for each AU.

Paw guarding was quantified using a cumulative pain score in mice with minor modifications^67^. Animals were placed in acrylic boxes with wire mesh floors and the incised hindpaw was closely observed during a 1-min period repeated every 5 min for 30 min. Depending on the position in which paw was found during the majority of the 1-min scoring period, a score of 0, 1, or 2 was given. Full weight bearing of the paw (score = 0) was present if the wound was blanched or distorted by the mesh. If the paw was completely off the mesh, a score of 2 was recorded. If the area of the wound touched the mesh without blanching or distorting, a score of 1 was given. The sum of the six scores (0–12) obtained during the 30 min session was plotted.

Thermal changes of the incised hindpaw and non-incised hindpaw were visualized using a FLIR T31030sc thermal imaging camera (FLIR instruments). Animals were placed in acrylic boxes with wire mesh floors and imaged in baseline conditions and 24 h post incision. Image analysis of the medial plantar surface was performed using the FLIR ResearchIR Max 4 software available at http://support.flir.com/rir4.

### Capsaicin-induced inflammatory pain model

Mice were habituated for 1 h to clear acrylic behavioral chambers before beginning the experiment. FLIR imaging and von Frey testing were performed using the methods described above. Thermal latency was measured using a Hargreaves device (IITC Life Science) with heated glass. Settings of 29 °C glass, 40% active laser power, and 20 s cut-off were used. CGRP receptor antagonist GGRP_8–37_ (1 μg, cat. # H-4924.0001, Bachem), scramble SPOT-ON (10 μg), or Poly(A) SPOT-ON (10 μg) were injected 15 min before intraplantar administration of 5 μg of capsaicin (cat. # M2028, Sigma). Mice were tested at 1, 3, and 24 h following intraplantar capsaicin administration. Drugs or capsaicin were injected with a volume of 10 μL via a 30.5-gauge needle. CGRP_8–37_, scrambled SPOT-ON, and Poly(A) SPOT-ON were diluted in 1× PBS. Capsaicin stock (1 mg/mL) was diluted in a solution 10% ethanol, 10% Tween-20, and 80% saline. At day 10, mice were assessed again before and after intraplantar injection of PGE_2_ (100 ng).

### Animal usage

All procedures that involved use of animals were approved by the Institutional Animal Care and Use Committee of The University of Texas at Dallas and were in accordance with International Association for the Study of Pain guidelines. All behavioral studies were conducted using male Swiss Webster (Taconic Laboratories) mice weighing between 20 and 25 g. Animals were housed with a 12-h light/dark cycle and had food and water available ad libitum. The experimenters measuring mechanical withdrawal thresholds, paw guarding, and facial expressions were blinded to the experimental conditions. Mice were randomized to groups from multiple cages to avoid using mice from experimental groups that were cohabitating.

### Transgenic mouse lines

To genetically label Schwann cells, mice that express Cre recombinase under the control of the myelin protein zero (*MPz*) gene^69^ were crossed with mice that have a loxP-flanked STOP fragment placed upstream of an enhanced green fluorescent protein fused to ribosomal protein unit L10a^70^. Mice were purchased from the Jackson Laboratory.

### Statistical analysis

In vitro data were collected from three independent cell culture wells and are shown as means ± s.d. or means ± s.e.m. In vivo (behavior) data are shown as means ± s.e.m. of six animals per group. Sample size was estimated as *n* = 5 using G*power for a power calculation with 80% power, expectations of 50% effect size, with *α* set to 0.05. Graph plotting and statistical analysis used GraphPad Prism Version 7.0 (GraphPad Software). Statistical evaluation was performed by one-way or two-way analysis of variance, followed by post hoc Bonferroni test, and the a priori level of significance at 95% confidence level was considered at *P* < 0.05. Student's *t* test was used to compare two independent groups. Specific statistical tests used are described in figure legends.

### Data availability

The data that support the findings of this study are available from the corresponding author upon reasonable request.

## Electronic supplementary material

Supplementary Information
