# Supplementary material for: Inhibition of Poly(A)-binding protein with a synthetic RNA mimic reduces pain sensitization in mice
Source: Nat Commun. 2018 Jan 2;9:10. doi: 10.1038/s41467-017-02449-5 (PMC5750225; doi:10.1038/s41467-017-02449-5)
Supplement: Supplementary file 1 — Supplementary Information [file 41467_2017_2449_MOESM1_ESM.pdf]

a

PABP expression in the DRG

Species	Gene	FPKM[1]
Human	PAPC1	124
Human	PAPC4	29
Human	PAPC3	0.03
Mouse	PAPC1	20
Mouse	PAPC4	3.5
Mouse	PAPC3	0.01

[1]Fragments per kilobase mapped (FPKM) normalized to the expression level of the upper quartile

b

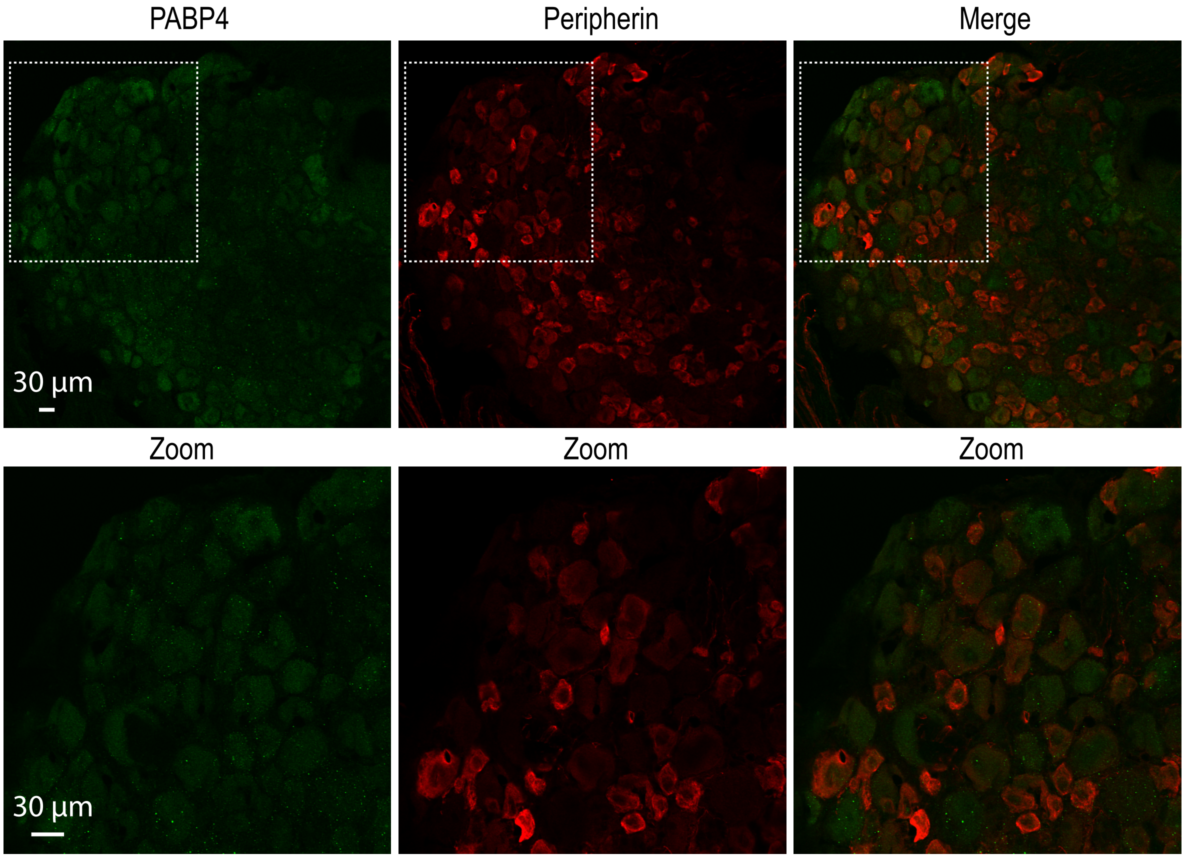

Supplementary figure 1. Expression and immunoreactivity of PABP4 in the DRG. (a) Expression of PABP isoforms in DRG of mouse and humans based on high-throughput sequencing. (b) PABP4, the second most abundant isoform in the DRG, fails to show a clear immunoreactivity in the DRG.

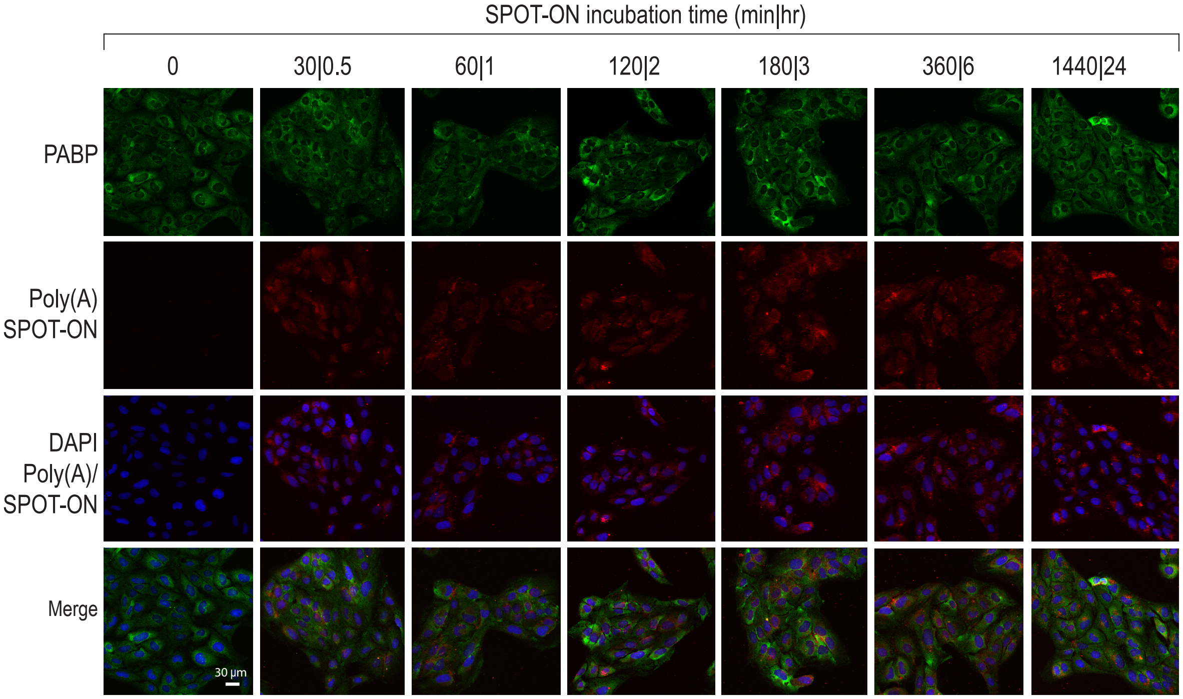

Supplementary figure 2. Time course showing the cellular uptake of SPOT-ONs. Cy-3 labeled Poly(A) SPOT-ONs are stable, efficiently taken up and distributed throughout the U2OS cells. Time course of quantification is shown in figure 2g.

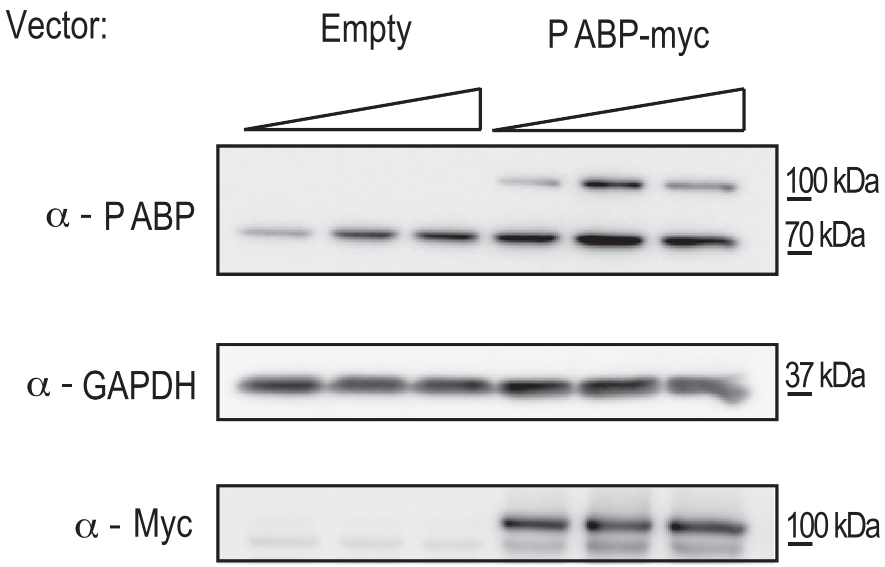

Supplementary figure 3. Overexpression of PABP confirmed by immunoblot. Antibodies to PABP, GAPDH, and Myc are arranged from Top to bottom. Three concentrations of vector (0.5, 1, and 2 μg) were transfected as before and expressed for 48 hours. Recombinant PABP was detected with a myc epitope tag.

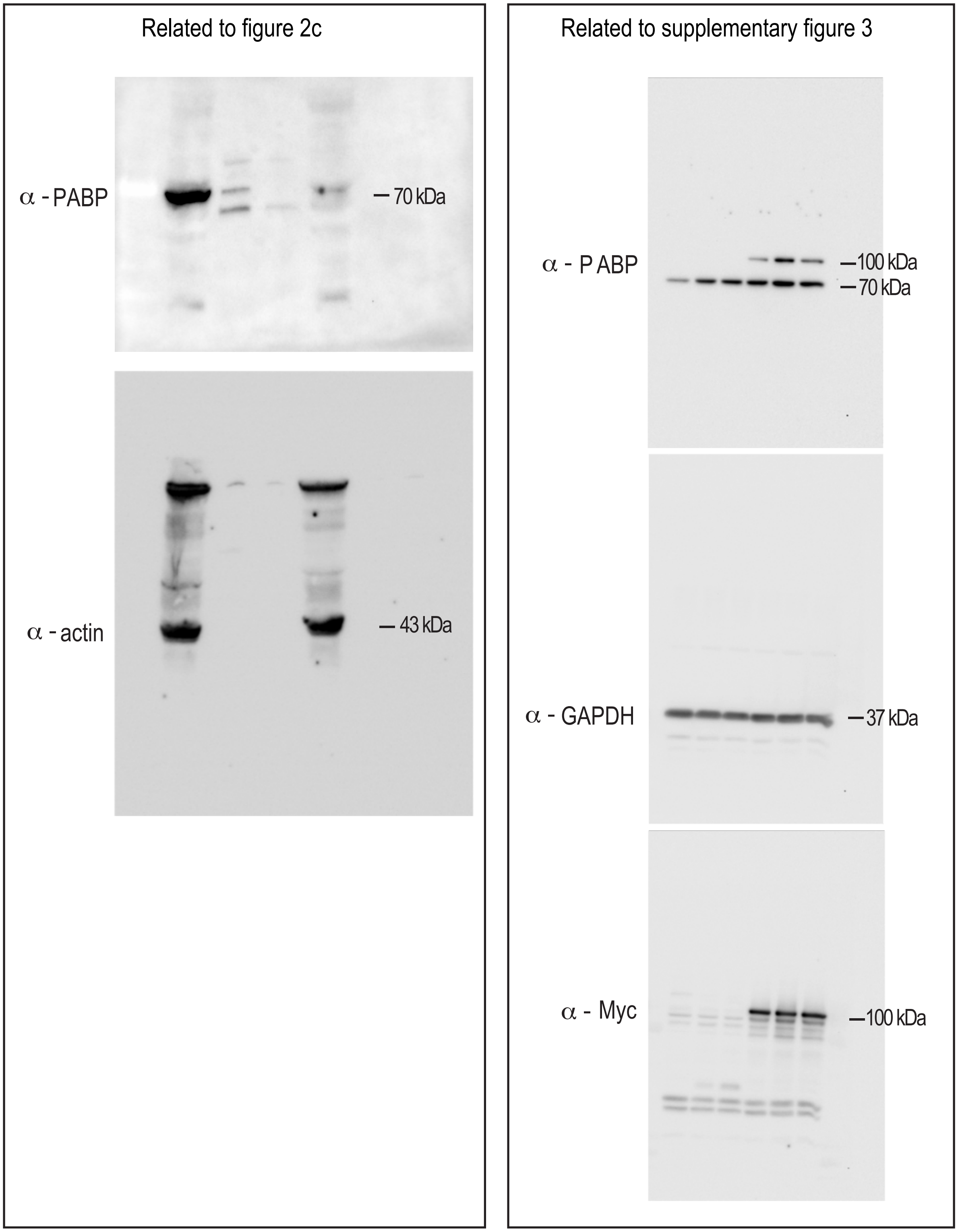

Supplementary figure 4. Full blots related to figure 2c and supplementary figure 3.
